# Use of daptomycin in the treatment of vancomycin-resistant enterococcal urinary tract infections: a short case series

**DOI:** 10.1186/1471-2490-13-33

**Published:** 2013-07-16

**Authors:** Divya Pradeep Ramaswamy, Maria Amodio-Groton, Stephen J Scholand

**Affiliations:** 1 Department of Internal Medicine, St. Mary’s Hospital, Waterbury, Connecticut 06706, USA; 2 Cubist Pharmaceuticals, Inc., Lexington, Massachusetts 02421, USA; 3 Division of Infectious Diseases, St. Mary’s Hospital, Waterbury, Connecticut 06706, USA; 4 Current affiliations: Toledo Hospital, Toledo, Ohio 43606, USA; 5 Current affiliations: Cornell Scott Hill Health Center, New Haven, Connecticut 06519, USA

**Keywords:** Daptomycin, *Enterococcus faecalis*, *Enterococcus faecium*, Urinary tract infection, Vancomycin-resistant enterococci

## Abstract

**Background:**

Vancomycin-resistant enterococci are a leading cause of hospital-acquired urinary tract infection and a growing concern for the clinician. The aim of this study was to evaluate the effectiveness of daptomycin in the treatment of patients with vancomycin-resistant enterococcal urinary tract infection treated in our 200-bed community-based institution.

**Methods:**

Patients with confirmed symptomatic vancomycin-resistant enterococcal urinary tract infection identified by infectious disease consultation between January 1, 2007, and December 8, 2009, vancomycin-resistant enterococci–positive urine culture, and urinary symptoms and/or pyuria on urinalysis, and treated with daptomycin, were included in this case series. Daptomycin was generally administered at a planned dosage regimen of ≥5 mg/kg every 24 hours in patients with normal to moderately impaired kidney function or every 48 hours in patients with severe kidney disease. Microbiologic cure was defined as eradication of vancomycin-resistant enterococci in urine cultures taken after the completion of daptomycin treatment. Clinical cure was defined by symptom resolution, as assessed by the infectious disease clinician caring for the patient.

**Results:**

Included in this case series are 10 patients who received daptomycin for confirmed vancomycin-resistant enterococcal urinary tract infection. Patients had a history of extensive hospital stays. Chart review revealed that all levels of kidney function (3, 2, 3, and 2 patients with kidney disease classified as normal, mild, moderate, and severe/kidney failure, respectively) were represented in the sample and that patients with (n = 5) or without (n = 5) previous urinary tract infection and with (n = 3) or without (n = 7) Foley catheters were included. Treatment with daptomycin achieved clinical cure and vancomycin-resistant enterococcal eradication in all cases in this series.

**Conclusion:**

Treatment with daptomycin was well tolerated and effective in all patients in this series, regardless of renal function, history of urinary tract infection, or Foley catheter use. This study adds to emerging clinical evidence that daptomycin is a valuable treatment for vancomycin-resistant enterococcal urinary tract infection.

## Background

In the United States, the Gram-positive bacterium *Enterococcus* accounts for 12% of all cases of hospital-acquired infection and is most often implicated in urinary tract infections (UTIs) [1-3]. Recent data show that approximately 33% of all clinical enterococcal isolates in the United States are vancomycin-resistant enterococci (VRE) [2]. In North America, VRE are mainly derived from the species *Enterococcus faecium* (92.8%) and *Enterococcus faecalis* (6.7%) [4]. In the past decade, VRE have become increasingly involved in nosocomial infections in the United States [5,6], which has resulted in excessive morbidity, mortality, and health care costs [7]. Nearly 10% of all urinary enterococcal isolates in the United States are VRE; most of these are also *E. faecium* (88.4%) [3].

Because VRE, particularly *E. faecium* strains, exhibit resistance to many antimicrobials traditionally used to target vancomycin-susceptible isolates, the treatment of patients with VRE UTIs remains a challenge for the clinician [8]. Indeed, no drugs are approved by the US Food and Drug Administration (FDA) for the treatment of patients with VRE UTIs, and reliable clinical data about the optimal management of VRE UTIs are lacking in the scientific literature [7]. Among the therapeutic options to be considered are those that have demonstrated activity against VRE in vitro, including older agents such as doxycycline, fosfomycin, and nitrofurantoin and newer agents such as daptomycin, linezolid, quinupristin–dalfopristin, and tigecycline [3,7,9].

Daptomycin is a bactericidal cyclic lipopeptide approved by the FDA for the management of complicated skin and skin structure infections caused by susceptible isolates of a variety of Gram-positive bacteria, including methicillin-resistant *Staphylococcus aureus*, and for the management of bacteremia, including that associated with right-sided infective endocarditis, caused by methicillin-susceptible and methicillin-resistant isolates of *S. aureus*[10]. Although the approved use of daptomycin in enterococcal infections is limited to the treatment of patients with complicated skin and skin structure infections caused by vancomycin-susceptible isolates of *E. faecalis*[10], 100% and 99.7% of vancomycin-resistant *E. faecalis* and *E. faecium*, respectively, were susceptible to daptomycin in more than 700 nonurinary VRE strains collected in the United States between 2007 and 2008 [11]. The daptomycin minimal inhibitory concentration required for 90% inhibition (MIC_90_) of vancomycin-resistant *E. faecalis* was determined to be 1 μg/mL, whereas that for vancomycin-resistant *E. faecium* was 2 μg/mL [11]. The use of daptomycin is a particularly promising pharmacotherapeutic approach against VRE UTIs because 50% to 70% of the dose is excreted unchanged in the urine 24 hours after intravenous administration compared with 30% to 40% for linezolid, 15% to 19% for quinupristin–dalfopristin, and 20% to 30% for tigecycline [7]. Moreover, another case series has described the successful use of daptomycin in patients with VRE UTIs [12].

In light of the scarcity of treatment options for VRE UTI and clinical data related to daptomycin use in this setting, the purpose of this report is to describe the clinical experience with daptomycin as part of the management of VRE UTIs in our acute care hospital. Our objective is to offer further evidence supporting daptomycin as a viable approach to managing a continuing therapeutic challenge.

## Methods

This study was conducted at St. Mary’s Hospital, a small community-based institution with approximately 200 beds, serving an urban population in Waterbury, Connecticut. Our catchment area included a number of nursing homes that contributed up to one-third of our inpatients. On request, one of the authors (SJS) provided expertise in the management of infectious diseases (ID). The current retrospective case series includes patients for whom ID consultation was requested over a 3-year period (from January 1, 2007, to December 8, 2009) and who ultimately received daptomycin for treatment of symptomatic VRE UTI. In summarizing the cases we managed for this report, we sought approval from the ethics committee of our institution (“the Saint Mary’s Hospital IRB”) (protocol 12-18-09). After IRB permission was granted, written informed consent was obtained from all patients for inclusion in this study and for publication of their medical information. A copy of the written consent is available for review by the editor of this journal.

A large majority of VRE-infected patients in our hospital are seen by an ID specialist because of intrinsic limitations in the treatment options and the hospital requirement of gown-and-glove contact isolation procedures for these patients. For this retrospective case series, patients were identified for consultation after a telephone call about a VRE-positive urine culture. To be treated with daptomycin and included in this analysis, patients had to exhibit urinary symptoms, pyuria (>5 white blood cells per high-power field), or both on urinalysis with a positive VRE culture.

VRE susceptibility was determined according to a VITEK 2 (bioMérieux, Inc, Durham, NC) microbial identification system, with daptomycin susceptibility determined by Etest (bioMérieux, Inc) on request. Patients who had positive VRE urinary cultures without significant pyuria on urinalysis were considered *colonized* rather than *infected* and, therefore, were not included in this analysis.

Once a patient was determined to have a VRE UTI, screening creatinine values were measured in all cases, and creatine phosphokinase (CPK) levels were determined in most cases. Daptomycin was generally administered at a planned dosage regimen of ≥5 mg/kg every 24 hours (if creatinine clearance [CrCl] ≥30 mL/min) or every 48 hours (if CrCl <30 mL/min). Dosage was determined based on actual body weight for all patients in this analysis. A dose of ≥5 mg/kg was chosen to provide ample urinary concentrations of the drug.

Clinical cure was defined by the resolution of symptoms in the best judgment of the ID clinician involved in the care of the patient (SJS). These included urinary symptoms such as dysuria, urinary frequency, and changes in urine character. Other symptoms such as abdominal pain, fever, malaise, and anorexia were also monitored for improvement. Follow-up urinalysis and urine culture were typically performed at the end of the daptomycin treatment course. The presence or absence of pyuria in urinalysis at the end of treatment was not in itself a strict criterion for clinical cure because it might have resulted from bladder irritation, drugs, or other causes. Regardless of whether pyuria was initially present and remained present at the end of the treatment course, microbiologic data were examined to help determine whether microbiologic cure was achieved. Microbiologic cure was defined by the eradication of VRE in urine cultures taken after completion of the daptomycin treatment course.

## Results

Between January 1, 2007, and December 8, 2009, we identified 10 patients with VRE UTIs who were treated by daptomycin-based regimens at our institution. Baseline characteristics of these patients are shown in Table 1; all patients had cystitis. As is apparent from the medical histories, most of the VRE UTIs were acquired in the hospital. In addition, the patients had a variety of risk factors typical for VRE infections, such as prolonged hospital stays, multiple previous UTIs, uropathy including the requirement for a long-term Foley catheter, and multiple past courses of antibiotics [7].

**Table 1:** Selected baseline clinical characteristics of patients infected with VRE UTIs selected for daptomycin therapy

**Patient**	**Age (y)/Sex**	**Weight (kg)**	**Medical history**	**Previous UTIs**	**Foley catheter**	**Cr (mg/dL)/ CrCl (mL/min)**	**Urinalysis (WBC/hpf)**	**VRE isolate**	**VRE culture (CFU/mL)**	**Susceptibilities**
**Normal kidney function**										
1	72/F	67	Long hospital stay after surgery for large mucinous adenocarcinoma of the ovary; ADM: lethargy thought secondary to sedative overdose	No	No	0.6/90	40-50	*E. faecium*	50,000	R: AMP, LNZ, TET, VAN; S: GEN, QD, TIG
2	61/F	107	MS, neurogenic bladder; ADM: MS flare, *Pseudomonas* spp. UTI treated with CIP	Yes	Yes	0.8/100	60-65	*E. faecium*	30,000	R: PCN, TET, VAN; I: NFT; S: DAP
3	30/M	66	C5 quadriplegia, neurogenic bladder, recent UTIs caused by *Pseudomonas aeruginosa*; ADM: abdominal pain, cloudy urine, treated empirically with TOB	Yes	No	0.2/>120	>100	*E. faecalis*	100,000	R: TET, VAN; S: DAP, PCN
**Mild kidney disease (CKD 2)**										
4	70/F	66	Neurogenic bladder, nephrolithiasis; ADM: lethargy, hypotension	Yes	Yes	0.6/83	25-30	*E. faecium*	>100,000	R: AMP, PCN, VAN
5	67/F	109	ADM: acute on chronic respiratory failure due to CHF, hypoventilation secondary to obesity; initially required intubation and ventilation in the ICU, developed fever (101°F); initially unspecified *Enterococcus* infection treated with VAN and CEF	No	No	1.4/63	NP	*E. faecium*	5000	R: AMP, NFT, VAN
**Moderate kidney disease (CKD 3)**										
6	83/F	55	Acute exacerbation of COPD	No	No	1.1/34	TNTC	*E. faecium*	>100,000	R: PCN, TET, VAN; I: NFT
7	83/F	77	Stays at a nursing home; recently hospitalized for stenting of right femoral artery for vascular disease	No	No	1.2/46	25-50	*E. faecium*	40,000	R: NFT, PCN, TET, VAN; S: DAP
8	86/M	70	CKD stage 3, nephrolithiasis, obstructive uropathy with benign prostatic hypertrophy, recent UTI caused by *E. coli* and *P. aeruginosa*; ADM: bloody urine, initial evaluation revealed infection with *E. coli* and *P. aeruginosa*, treated with CEF	Yes	Yes	1.1/43	10-15	*E. faecium*	80,000	R: PCN, TET, VAN
**Severe kidney disease/kidney failure (CKD 4–5)**										
9	90/F	60	Severe dementia, stays at extended-care facility; ADM: decreased oral intake, abdominal pain, fever (100.9°F)	Yes	No	1.1/26	TNTC	*E. faecium*	>100,000	R: AMP, CIP, NFT, TET, VAN
10	60/M	81	Liver transplant for hepatitis C cirrhosis; ADM: chest pain, *E. coli* empyema complicating previous CABG surgery	No	No	4.1/19	NP	*E. faecium*	100,000	R: DOX, PCN, VAN

*ADM* admission, *AMP* ampicillin, *CABG* coronary artery bypass graft, *CEF* ceftazidime, *CFU* colony-forming units, *CHF* congestive heart failure, *CIP* ciprofloxacin, *CKD* chronic kidney disease, *COPD* chronic obstructive pulmonary disease, *Cr* creatinine, *CrCl* creatinine clearance, *DAP* daptomycin, *DOX* doxycycline, *GEN* gentamicin, *I* intermediate, *ICU* intensive care unit, *LNZ* linezolid, *MS* multiple sclerosis, *NFT* nitrofurantoin, *NP* not performed, *PCN* penicillin, *QD* quinupristin–dalfopristin, *R* resistant, *S* susceptible, *TET* tetracycline, *TIG* tigecycline, *TNTC* too numerous to count, *TOB* tobramycin, *UTI* urinary tract infection, *VAN* vancomycin, *WBC/hpf* white blood cells per high-power field.

Table 2 presents the details of the daptomycin-based regimens for the management of VRE UTIs in our selected patient cases. Dosages used were based on our empirical experience because there are no recommendations in the literature with regard to daptomycin use in the treatment of patients with VRE UTIs. Because of the high therapeutic index and the fixed vial size for daptomycin (500 mg), some variability in actual per-weight dosing occurred. The unusually high dose used in patient 3 (13 mg/kg every 24 hours in a 30-year-old man with quadriplegia) is explained by the concomitant management of a *Staphylococcus* spp. bacteremia.

**Table 2:** Details of management of VRE UTIs and outcome of daptomycin course

**Patient**	**Daptomycin dosing**	**Daptomycin treatment duration**	**Concomitant antibiotics**	**Posttreatment urine culture**
**Normal kidney function**				
1	7.5 mg/kg q24h	7 days	None	VRE eradicated
2	8 mg/kg q24h	3 days	None	VRE eradicated
3	13 mg/kg q24h*	10 days	None	VRE eradicated
**Mild kidney disease (CKD 2)**				
4	5 mg/kg q24h	3 days	Fluconazole	VRE eradicated
5	5 mg/kg q24h	3 days	None	VRE eradicated
**Moderate kidney disease (CKD 3)**				
6	5 mg/kg q24h	3 days	None	VRE eradicated
7	5 mg/kg q24h	3 days	Gentamicin 120 mg once a day	5 days after initiation of daptomycin; VRE eradicated
8	5 mg/kg q24h	3 days	None	VRE eradicated
**Severe kidney disease/kidney failure (CKD 4–5)**				
9	5 mg/kg q24h^†^	3 days	None	VRE eradicated
10	5 mg/kg q48h	3 days	None	VRE eradicated; patient died 2 days later (not related to infection)

*CKD* chronic kidney disease, *q24h* once every 24 hours, *q48h* once every 48 hours, *VRE* vancomycin-resistant enterococci.

As determined using the Cockcroft-Gault equation, CKD 2 = glomerular filtration rate (GFR) of 60–89 mL/min/1.73 m^2^, CKD 3 = GFR of 30–59 mL/min/1.73 m^2^, and CKD 4–5 = GFR of <30 mL/min/1.73 m^2^.

Follow-up durations: microbiologic assessment (urine culture) ~3 days, clinical assessments up to ~7 days posttreatment.

*Use of unusually high per-weight dose was recommended by the ID specialist for the concomitant management of *Staphylococcus* spp. bacteremia.

^†^ID consult resulted in clinical decision to treat q24h rather than q48h for more rapid results in this patient.

Daptomycin-based courses of antibiotic treatment achieved clinical cure and successful eradication of VRE in all patients in this representative sample of a diverse patient population in our medical facility (Table 2). Indwelling catheters are common sites of infection, and similar results of daptomycin treatment were observed in patients with and without Foley catheters. Daptomycin was effective at eradicating VRE in patients regardless of whether they had had previous UTIs. Additionally, daptomycin eradicated VRE regardless of the level of renal function. Overall, daptomycin was well tolerated, and no reports of adverse events such as alteration of kidney function, muscle weakness or pain, and elevated levels of CPK enzymes were included. For most patients, CPK levels were measured at the time of daptomycin therapy initiation and once or twice more in the next 7 to 10 days. Even for the patient receiving the highest dose (13 mg/kg, patient 3), no elevation was above the normal CPK range.

## Discussion

The data in this case series show daptomycin to be a safe and effective therapeutic option in the treatment of patients with VRE UTIs. Limitations of the present study include the fact that this is a retrospective case series with a small number of patients and clinical records that are often limited and variable. Furthermore, because of the paucity of data in the literature, it is unclear what the most appropriate daptomycin dose is for the treatment of VRE UTIs. Nevertheless, it is clear that VRE are increasingly involved in nosocomial infections. Of concern, VRE may transfer vancomycin resistance to other bacterial species, including *S. aureus*[13]. Uncontrolled dissemination of VRE infections within health care institutions has been facilitated by incautious contact with contaminated medical equipment and surfaces, colonized health care personnel, and infected patients [14]. For example, the spread of a strain of linezolid-resistant VRE has been reported in one zone of a transplantation unit despite extensive precautions [15].

Another major factor that has led to the spread of VRE infection is poor infection control techniques that rely on cephalosporins and other antibacterials inactive against enterococci [8]. A recent report [7] found that more than 30% of all clinical isolates of *Enterococcus* were resistant to vancomycin, including more than 90% of *E. faecium* isolates. For these reasons, appropriate antibiotic susceptibility testing is a mainstay of good clinical practice to limit the spread of multidrug-resistant strains, and agents that have specific activity against these strains must be used to eradicate these difficult-to-treat infections.

The urinary tract is one of the main portals for entry of VRE, so it is hardly surprising that the urinary tract is a major site of infection [13]. Management of VRE UTIs usually requires correction of any factors contributing to the infection. Catheter removal, obstruction relief, abscess drainage, and initiation of antimicrobial therapy are all initial steps taken in clinical practice [7].

In this case series, patients with VRE UTIs were treated with daptomycin, a cyclic lipopeptide antibiotic that has rapid bactericidal activity against a variety of Gram-positive pathogens [16]. Daptomycin acts by binding to bacterial cell membranes and inducing rapid depolarization, which inhibits DNA, RNA, and protein synthesis and leads to cell death [10]. In the 10 cases described here, daptomycin was chosen as the treatment option because of its efficacy profile, sensitivity testing, and low resistance rates for both major species of *Enterococcus*.

Another important consideration when appropriate treatment for VRE UTI is chosen is the presence of intact drug at the site of infection [16]. Daptomycin is eliminated primarily by the kidney; approximately 52% is excreted unchanged into the urine after intravenous administration [10]. Other agents active against VRE, such as linezolid, quinupristin–dalfopristin, and tigecycline, have a lower fraction of urinary excretion [7], which may potentially limit their effectiveness in the management of VRE UTIs.

Most strains of VRE are resistant to penicillin and ampicillin, although higher-dose ampicillin, doxycycline, and nitrofurantoin remain viable treatment options. More often, drug choices used to treat VRE include daptomycin, linezolid, quinupristin–dalfopristin, and tigecycline. These compounds have similarly low MIC values against VRE species [7]. All these antibiotics except daptomycin exhibit bacteriostatic properties; daptomycin is bactericidal [7]. Daptomycin has similar MIC values for *E. faecium* and *E. faecalis*[7] and is effective in treating either pathogen. Daptomycin resistance among VRE strains remains rare [11]; numerous global surveillance studies have demonstrated higher susceptibility levels in VRE strains using daptomycin than in strains using linezolid or quinupristin–dalfopristin [16]. Resistance to linezolid among VRE isolates has been well described and is of increasing clinical concern [16-19]. Quinupristin–dalfopristin has limited activity against *E. faecalis*[7]. In addition, vancomycin-resistant *E. faecium* strains have shown emerging resistance to quinupristin–dalfopristin; 3.4% of urinary tract isolates were resistant in a study at 28 medical centers in the United States [3]. Use of quinupristin–dalfopristin is also limited by its potential systemic and infusion site–related adverse effects, including myalgia and arthralgia [7,13].

Our experience confirms and extends the findings of a previous case series on the use of daptomycin for the treatment of VRE UTIs. The previous study [12] of 5 hospitalized patients with indwelling catheters and VRE UTIs who received 5-day courses of daptomycin showed that all patients achieved complete eradication of infection at daptomycin doses of 1.4 to 3.7 mg/kg daily. Taken together, these case studies emphasize the importance of further analyses to delineate the appropriate doses of daptomycin for the treatment of patients with VRE UTIs.

## Conclusions

In the current case series, daptomycin was shown to be a safe and effective therapeutic option in the management of VRE UTIs. Because of the increasing prevalence of VRE infection and the limited treatment options available, we anticipate that management of VRE UTIs will continue to be challenging for the clinician. Based on our experience, we believe that daptomycin is a valuable treatment option for problematic UTIs. These findings must be confirmed in larger randomized clinical trials. However, despite the lack of data about therapeutic options for VRE UTIs and the intrinsic limitations of descriptive case reports, our positive experience with daptomycin may be of value to the clinical community.

## Abbreviations

CPK: Creatinine phosphokinase; CrCl: Creatinine clearance; FDA: Food and Drug Administration; ID: Infectious disease; IRB: Institutional review board; MIC: Minimal inhibitory concentration; UTI: Urinary tract infection; VRE: Vancomycin-resistant enterococci.

## Competing interests

DPR and SJS have no competing interests to report. MA-G is an employee of Cubist Pharmaceuticals, Inc. The authors received no financial support or honoraria for the development of this manuscript.

## Authors’ contributions

DPR and SJS conceived of the study, collected and analyzed data from patient records, wrote the first draft of the manuscript, and reviewed all subsequent drafts. MA-G contributed to data analysis and interpretation and reviewed all drafts. All authors read and approved the final manuscript.

## Pre-publication history

The pre-publication history for this paper can be accessed here:

http://www.biomedcentral.com/1471-2490/13/33/prepub
